# Detection of *Dirofilaria repens* and *Mansonella llewellyni* in the United States by *Wolbachia* Surveillance

**DOI:** 10.1155/tbed/2778610

**Published:** 2025-07-21

**Authors:** Charlotte O. Moore, Cynthia Robveille, Barbara Qurollo, Edward B. Breitschwerdt

**Affiliations:** Department of Clinical Sciences, College of Veterinary Medicine, North Carolina State University, Raleigh, North Carolina, USA

**Keywords:** *Dirofilaria immitis*, *Dirofilaria* spp., dog, phylogenetic, raccoon

## Abstract

In mammals, detection of *Wolbachia* bacteria can be used to diagnose filarial infection, while antibiotic treatment to eliminate *Wolbachia* can assist in eliminating filarial infections. Because *Wolbachia* are necessary for survival of several filarioids and closely related to *Anaplasma* and *Ehrlichia*, we analyzed *Wolbachia* DNA amplification by *Anaplasma/Ehrlichia* qPCR, from 39,526 domestic and wildlife animal blood samples submitted to a diagnostic laboratory between 2017 and 2023. Filarial infection was confirmed by 28S gene amplification, followed by phylogenetic analysis utilizing filarial cytochrome oxidase subunit 1 (*cox1*), myosin heavy chain (*myoHC*), and 70 kilodalton heat shock protein (*hsp70*) gene sequencing. *Wolbachia* DNA was detected in 57 domestic dogs (*Canis familiaris*) and three raccoons (*Procyon lotor*) from 23 states and Puerto Rico. A majority of the *Wolbachia* sequences from dogs were *Dirofilaria immitis*-associated (89%, 51/57), whereas DNA from other *Wolbachia* were associated with insects (9%, 5/57) or *Dirofilaria repens* (2%, 1/57). *D. immitis* infection was confirmed by 28S filarial PCR for all samples with *D. immitis*-associated *Wolbachia* available for testing (*n* = 41). *D. repens* infection was confirmed by 28S and *cox1* PCR in the dog infected with *D. repens*-associated *Wolbachia*. This dog was originally imported from Slovakia. The *Wolbachia* DNA amplified from raccoons most closely aligned with *Wolbachia* from *Mansonella ozzardi* (98.9%). 28S filarial, *cox1*, *myoHC*, and *hsp70* sequencing did not align with currently available GenBank sequences but did align with *Mansonella*. Morphologically, microfilariae from additional raccoons were consistent with *Mansonella llewellyni*. Molecular surveillance for *Wolbachia* in wildlife and domestic animals has the potential to identify novel filarial species in the United States, including zoonotic species.

## 1. Introduction

The domestic dog is host to over 15 filarioid species associated with clinical manifestation of variable severity (Table 1) [3, 5–8]. Manifestations include pruritic lesions and nodules (*D. repens* [*Dirofilaria repens*] and *O. lupi* [*Onchocerca lupi*]), lymphatic obstruction (*Brugia* spp.), and cardiopulmonary symptoms (*D. immitis [Dirofilaria immitis]*) [1, 9]. These can be triggered by the physical obstruction of the adult worm, host immune activation, or even a life-threatening response to antimicrofilarial treatment [9, 10]. The most common canine filarial infection in the United States is *D. immitis*, the cause of canine heartworm disease.

Due to a lack of screening and the large number of dogs transported around the world, the risk of importing canine filaria species whose known range does not include the United States, such as *D. repens* or *Brugia* spp., is concerning considering the potential for zoonosis [11]. Furthermore, the vast majority of canine and feline filaria testing in the United States utilizes assays specific only for *D. immitis* antigens. Unlike the Modified Knott's Test (MKT), the *D. immitis* antigen test (SNAP 4Dx Plus, IDEXX, and WITNESS Canine Heartworm Antigen Test, Zoetis) only detects adult, female *D. immitis* antigens [12]. Due to this factor, the antigen test will only detect mature infections after approximately 6 months. Sensitivity to other filarioids is very low with occasional reports of cross-reactions to *Acanthocheilonema* or *D. repens*. Therefore, methods that are capable of broad detection of multiple filarioid species including those with limited known geographic range should be prioritized for surveillance.

Both *D. immitis* and *D. repens* thrive in areas where domestic dog populations do not receive preventative and high environmental temperature and humidity facilitate vector reproduction and parasite development. *Dirofilaria immitis* has a worldwide distribution while *D. repens* is associated with the “Old World” (Europe, Asia, and Africa) (reviewed by [13]). Two reports suggest *D. repens* is present or has been imported to the “New World” [14, 15]. In Europe, *D. repens* has spread at a faster rate than *D. immitis*, reflected in zoonotic and canine transmission [13]. Zoonotic transmission of *Dirofilaria* species can occur by the bite of an infected mosquito. In a majority of cases, infection is eliminated by the immune system however in atypical cases or immunocompromised individuals, infection may develop and even produce microfilaria. Larval *Dirofilaria* migrate through the body to a wide variety of body tissues including the skin, eyes, viscera (lungs and mesentery), muscle, lymph nodes, breasts, or male genitalia [2, 13]. In these patients, a dirofilariasis lesion can mimic tuberculosis, fungal infection, or neoplasia, resulting in the need for an excisional biopsy, a cause of costly interventions and emotional distress. Zoonotic *D. immitis* infection has been described on all continents except Antarctica. Serological surveys suggest *D. immitis* and *D. repens* exposure in the human population is similar, 1.5%–9.3%, with higher seroprevalences reported in areas where *Dirofilaria* are common in canine populations [16–18].

Additional domestic and wild animal species also host filarioids, including the raccoon (*Procyon lotor*). While the raccoon is host to a wide diversity of filarioids (*Brugia beaveri*, *Dracunculus insignias*, *Dipetalonema procyonis*, *Dirofilaria tenuis*, and *Mansonella llewellyni*) little research has addressed the epidemiology, pathology, or zoonotic capacity of these species [19–24]. *Mansonella llewellyni* is among the most prevalent of these species in the southeastern United States, however reports are restricted to geographically limited studies or case studies [22, 24–29]. Various filaria, including *Brugia*, *Dirofilaria*, *Mansonella*, and *Onchocerca* spp., are host to endosymbiotic bacteria in the genus *Wolbachia* [30, 31].

*Wolbachia* are obligate intracellular bacteria in the family Anaplasmataceae, closely related to *Anaplasma* and *Ehrlichia* spp. Elimination of this endosymbiont in infected filarioid species prevents microfilaria production and release and eventually results in premature adult filarioid death [32, 33]. *Wolbachia* also directly interacts with the mammalian host, as it is believed to drive neutrophil recruitment and activation, as well as, classic activation of macrophages [34, 35]. Given the association of *Wolbachia* and filaria, *Wolbachia* is considered a promising target for diagnosis and treatment of various filarioids including *Dirofilaria* and *Mansonella* species [36].

This retrospective study assessed *Wolbachia* detection in animal samples submitted to the North Carolina State University–Vector Borne Disease Diagnostics Laboratory (NCSU–VBDDL) for *Anaplasma* and *Ehrlichia* 16S qPCR, because of the close phylogenetic relationship of *Anaplasma*, *Ehrlichia*, and *Wolbachia*, the diagnostic *Anaplasma* and *Ehrlichia* 16S qPCR nonspecifically amplifies *Wolbachia* DNA. The first objective was to assess the mammalian host species and geographic location of *Wolbachia* infected animals, and to determine the filaria or arthropod species associated with the sequenced *Wolbachia*. The second objective was to amplify filarioid DNA from *Wolbachia* positive blood samples and phylogenetically compare non-*D. immitis* filaria by using three common gene targets: cytochrome oxidase subunit 1 (*cox1*), myosin heavy chain (*myoHC*), and 70 kilodalton heat shock protein (*hsp70*). Based on the detection of *Wolbachia* and an uncharacterized *Mansonella* filaria in raccoons in our study, and the lack of deposited *Mansonella* sequences from the United States in GenBank, the third objective was to further assess the phylogenetic identity and morphology of *Mansonella* spp. in the raccoons utilizing blood and tissue samples from additional animals.

## 2. Materials and Methods

### 2.1. Retrospective Testing

This retrospective study investigated the detection of *Wolbachia* spp. in animal blood samples submitted to the NCSU–VBDDL for *Anaplasma* and *Ehrlichia* qPCR from March 9, 2017 to December 31, 2023. As blood samples were submitted for diagnostic purposes from the animal's owner, ethical approval for animal use was not applicable. The authors have previously reported the nonspecific amplification of *Wolbachia* from *Ctenocephalides felis* utilizing these primers [37]. *Wolbachia* identity was considered definitive if an NCBI BLAST search returned a single alignment with > 99% homology to an arthropod or filaria associated *Wolbachia* [38].

*Dirofilaria immitis* antigen test results were obtained if performed by the NCSU–VBDDL (SNAP 4Dx Plus, IDEXX, Maine, USA). For the *D. immitis*-associated *Wolbachia* infected dogs, the accuracy, sensitivity, specificity, positive predictive value (PPV), and negative predictive value (NPV) of *D. immitis*-associated *Wolbachia* were all calculated in R (version 4.4.1) with the epiR package (version 2.0.80) utilizing SNAP 4Dx Plus results as the gold standard.

Blood stored at − 80°C or extracted DNA from blood was available for 50 animals (47 dogs and three raccoons) that were retrospectively identified as *Wolbachia* qPCR positive. DNA was extracted utilizing the Qiagen QIAsymphony SP robot with the QIAsymphony DSP DNA Mini Kit (Qiagen, Valencia, CA, USA; catalog no. 937236). Amplification by conventional PCR and sequencing of a portion of the 28S filarioid DNA was utilized to determine the presence and species of infecting filaria [39]. PCR cleanup and sequencing for all reactions was performed by GENEWIZ from Azenta Life Sciences (Azenta, Plainfield, NJ 07080, USA). Sequences were compared to GenBank utilizing NCBI BLAST and those with >99% homology to a single species were considered definitively identified. For *D. immitis*-associated *Wolbachia* infected dogs, PPV was calculated utilizing 28S filarioid PCR as the gold standard. This is because *Wolbachia* PCR negative dogs were not subject to 28S filarioid PCR, therefore only true positive and false positive samples could be identified.

For animals infected with an insect-associated *Wolbachia* species, or without detectable filarioid DNA, the submitting veterinarian was contacted to obtain medical records. Medical records were reviewed by one author (Edward B. Breitschwerdt) and key clinicopathologic findings were reported in the results. For animal(s) with invasive filarioid species, the owner was contacted to determine the animal's country of origin and current status.

To further define filarioid species not found in the United States or without a definitive identity, the *cox1*, *myoHC*, and *hsp70* filaria genes were amplified and sequenced [30, 40, 41]. Phylogenetic trees were constructed to compare sequences to reference sequences acquired from GenBank utilizing the RAxML (version 8.2.12) General Time Reversible model with gamma distribution for rate heterogeneity with 1000 bootstrap replicates [42].

### 2.2. Prospective Raccoon Sampling

Upon the initial amplification of filarioid sequences from raccoons that did not match publicly available sequences on GenBank, we collected samples from five additional raccoons on January 15, 2024 in Ellerbe, North Carolina with the assistance of a local trapper. Researchers only had contact with raccoons postmortem and animals were not trapped specifically for research purposes, therefore animal use approval was not necessary. Blood was obtained via postmortem cardiac blood draw and preserved in individual EDTA tubes. Four to seven skin snips were collected from each raccoon for incubation in sterile saline to identify adult filarioids.

Two hundred microliters of blood was aliquoted and DNA extracted as previously described utilizing the Qiagen QIAsymphony SP robot. PCR and sequencing of the *Wolbachia* 16S and 28S filarioid, *cox1*, *myoHC*, and *hsp70* genes were performed for comparison to filarioid sequences obtained from raccoons identified by the NCSU–VBDDL [30, 39, 40, 43] (Table S1).

For each racoon, five thin blood smears were prepared with 10 µL of blood, air-dried, and stained with either Diff-Quick or Wright-Giemsa. One or more MKT were performed between 126 and 357 days after blood collection for four raccoons with adequate residual blood [44]. All the slides were evaluated by a board-certified pathologist (Cynthia Robveille) in a blinded manner.

To measure microfilaria total length and maximum diameter, 48 microfilariae from four stained blood smear slides from three raccoons and 57 microfilariae from four MKT slides from two raccoons were analyzed. Slides were scanned with the Leica Aperio AT2 by the NCSU College of Veterinary Medicine Histology Laboratory and were analyzed by one author (Cynthia Robveille) using SlideViewer (3DHISTECH Kft). 95% confidence intervals for length and maximum diameter, as well as Wilcoxon rank sum test comparing length and maximum diameter by slide preparation technique (MKT or blood smear), were calculated utilizing the crosstable package (version 0.8.1) in R [45]. Three microfilaria features (head space, nerve ring, and excretory pore) were measured on the MKT slides and reported as a percentage of total body length from the anterior end.

Using the same technique described above, we measured the total length of 15 *Mansonella llewellyni* microfilariae from a Giemsa-stained thick blood smear slide generated by a previous publication for submission to the Harold W. Manter Laboratory of Parasitology collection [24]. The reference slide (HWML38947) is originally from Georgia, USA.

## 3. Results

### 3.1. Diagnostic Screening

**Figure 1:**
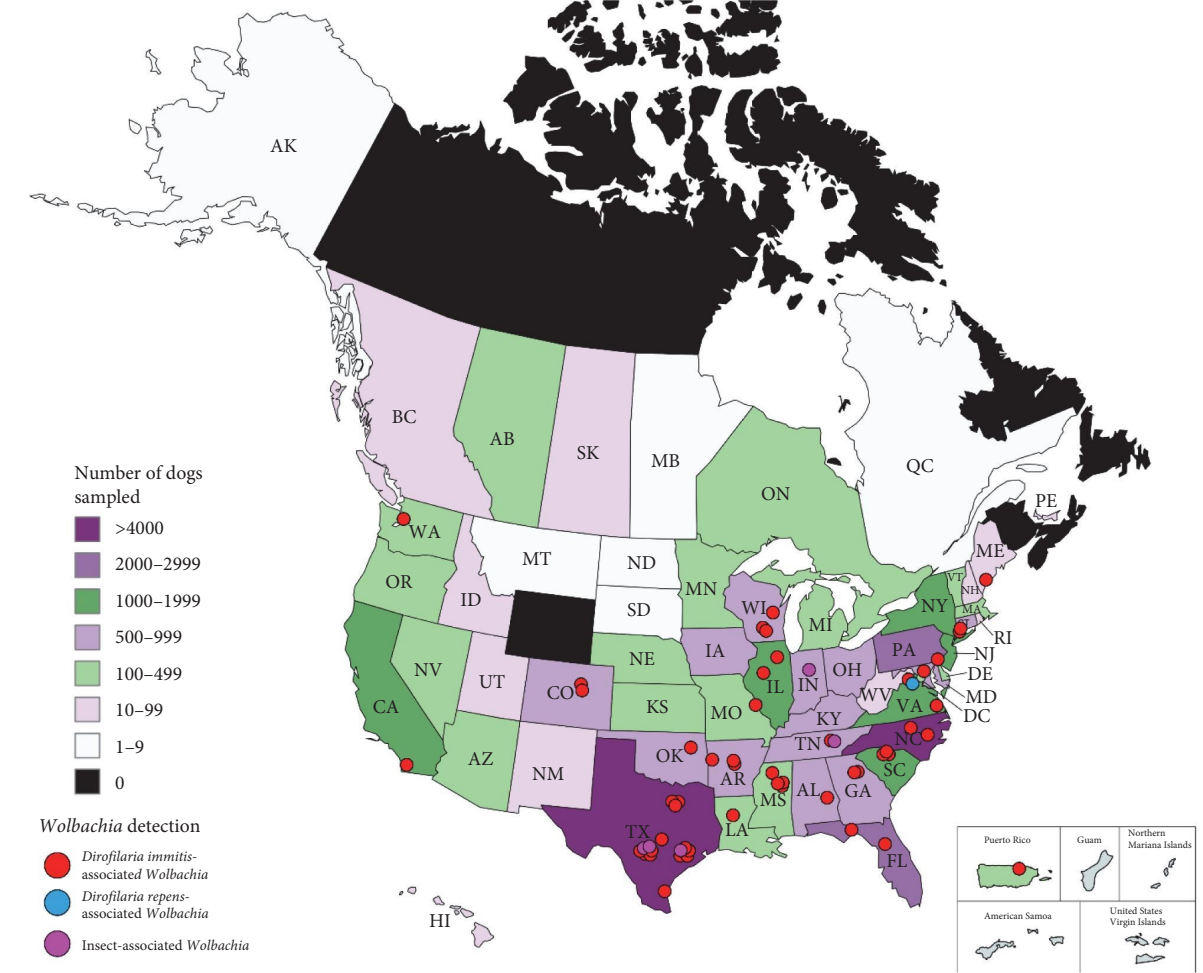
Distribution of sampling and location of *Wolbachia* 16S qPCR positive dogs from the samples submitted to the NCSU–VBDDL for *Anaplasma/Ehrlichia* 16S rRNA qPCR.

In total, 39,526 samples were submitted to the NCSU–VBDDL during study enrollment from at least 45 domestic and wild animal species (Table S2). Eight samples were from an unknown animal species, often with a group or family designation (e.g., unknown primate). Approximately 98% of samples were from domestic dogs (*n* = 35,179) or cats (*n* = 3,666). In total, *Wolbachia* DNA was detected in 60 samples from 23 states and Puerto Rico including raccoons (3/40; 7.50%) and domestic dogs (57/35,179; 0.16%; Figure 1). All raccoon samples tested by the NCSU–VBDDL, infected or uninfected, were from Kentucky.

### 3.2. Domestic Dogs

Based on alignment of the amplified portion of the 16S gene, *Dirofilaria immitis*-associated *Wolbachia* was the most frequent *Wolbachia* in domestic dogs (51/57, 89.5%; GenBank Accession ID CP046578.1, 180/180 bp, 100%). *Dirofilaria repens*-associated *Wolbachia* (AJ276500.1, 100%) was detected in a single dog.

*Wolbachia* associated with insect species were detected in five dogs (5/57, 8.8%) (Table 2). This included *Wolbachia pipientis* (CP092141.1, 181/181 bp, 100%) in two dogs, *C. felis*-associated *Wolbachia* (CP116767.1, 181/181 bp, 100%) in a single dog, and two insect-associated *Wolbachia* with multiple alignments to Supergroup A *Wolbachia* (OZ187006.1, 189/190 bp, 99.5% for the 5-year-old dog; OX366347.1, 181/181 bp, 100% for the 10-year-old dog). Their demographic and clinical presentations were broad (Table 2).

Compared to 4Dx SNAP Plus results generated by the NCSU–VBDDL, a majority of *D. immitis*-associated *Wolbachia* 16S qPCR positive dogs were *D. immitis* antigen positive (37/42, 88%), while five (12%) were antigen negative and nine were not tested (Table 3). Analyzing the 23,085 dogs tested by both 4Dx SNAP Plus (gold standard) and *Wolbachia* 16S qPCR, detection of *D. immitis*-associated *Wolbachia* by 16S qPCR boasted high specificity (100%) and NPV (99%) but limited sensitivity (18%; Table 4). The one *D. repens*-associated *Wolbachia* positive and five insect-associated *Wolbachia* 16S qPCR negative dogs were all 4Dx SNAP Plus *D. immitis* antigen negative. From medical records, one insect-associated *Wolbachia* dog tested 4Dx SNAP Plus positive for *D. immitis* antigens at their home clinic (2 days prior to sample submission) and *D. immitis* were found during autopsy 23 days later. Evidence of *D. immitis* infection was not reported in the records for the other insect-associated *Wolbachia* infected dogs.

Previously extracted DNA for additional analysis was acquired for 47 dogs. 28S filarial PCR amplified *D. immitis* DNA from all dogs with *D. immitis*-associated *Wolbachia* (41/41, 100%). Therefore, when utilizing 28S filarial PCR as the gold standard, detection of *D. immitis*-associated *Wolbachia* had a 100% PPV (91%–100%; 95% CI) (Table 4). *Dirofilaria repens* DNA was amplified from the one dog with *D. repens*-associated *Wolbachia*. Despite triplicate PCR, no filarial DNA was amplified from the five dog blood samples containing insect-associated *Wolbachia* (*W. pipientis*, *C. felis*, and nonspecific insect *Wolbachia*).

The dog infected with *D. repens* was originally imported to Virginia, USA from Slovakia but was unfortunately lost to follow up. Due to limited sample remaining, only the *cox1* filarial gene was amplified. The sequence aligned to *D. repens* (100%, 655/655 bp; MT345575.1) and was deposited in GenBank (Accession ID PV258722).

### 3.3. Raccoons

The *Wolbachia* detected in three raccoons submitted to the NCSU–VBDDL were all most closely aligned with *Mansonella ozzardi*-associated *Wolbachia* (AJ279034.1, 179/181 bp, 98.9%). Sequences from 28S filarial PCR aligned 100% with both *M. ozzardi* (GenBank Accession ID MN432519.1) and *Mansonella perstans* (MN432520.1).

**Figure 2:**
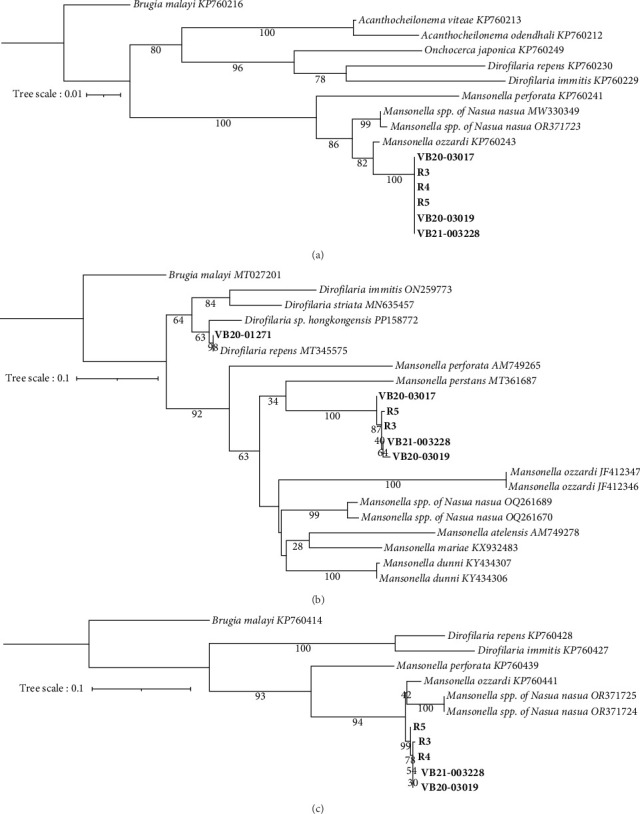
Alignment of non-*D. immitis* filaria to known sequences. Sequences generated in this manuscript are shown in bold while previously published species are in italics. Phylogenetic trees were generated for (A) *myoHC* (570 bp), (B) *cox1* (570 bp), and (C) *hsp70* (468 bp).

Prospective sampling identified an additional three (3/5, 60%) *Wolbachia* spp. qPCR positive raccoons from Ellerbe, NC. No adult filaria were identified from skin snips. All *Wolbachia* qPCR positive raccoons, but not the *Wolbachia* spp. negative racoons, were 28S filarial PCR positive with the same *Mansonella* as raccoons tested by the NCSU–VBDDL. Additional sequencing targeting the *cox1* (570 bp), *myoHC* (517 bp), and *hsp70* (468bp) genes was performed for comparison with a wide variety of filarial species (Figure 2). The amplified *Mansonella* spp. was distinct from human-associated *Mansonella* (*M. ozzardi*, *M. perstans*, and *Mansonella streptocerca*) and *Mansonella* previously reported in *Nasua nasua* (ring-tailed coati) in Brazil [41]. The sequences from raccoon filaria were deposited in GenBank: *cox1* (PV258720-1, PV258723-5), *myoHC* (PV269753-8), and *hsp70* (PV299582-6).

**Figure 3:**
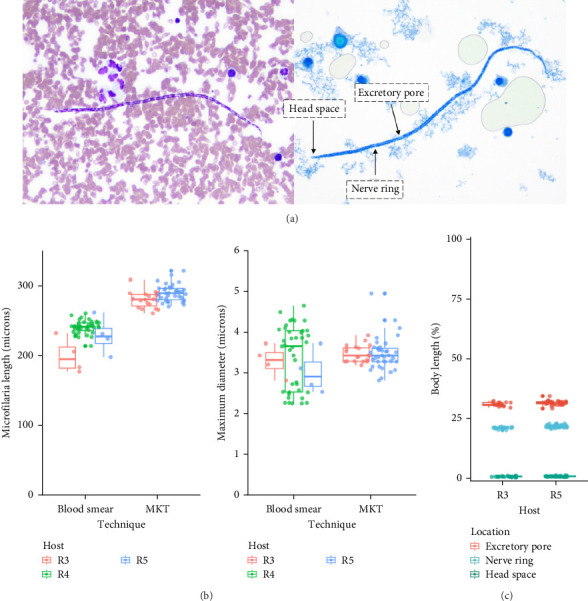
*Mansonella llewellyni* microfilaria obtained from *Procyon lotor*. (A) Images of microfilaria from stained blood smear and Modified Knott's Test (MKT). The location of microfilaria features measured on the MKT are labeled. (B) Total length and maximum diameter of microfilariae from three different raccoons (R3–R5) utilizing two visualization techniques (blood smear and MKT). (C) Location of three features (excretory pore, nerve ring, and head space) on MKT microfilariae expressed as a percentage of body length.

Microfilariae were observed on stained blood smears and MKT in the three PCR positive raccoons, but not in the two PCR negative raccoons (Figure 3A). The length of the microfilariae (Figure 3B) was significantly shorter (*p* < 0.0001) on the stained thin blood smears (236 ± 5 µm; mean ± 95% CI) compared to the MKT (287 ± 3 µm). Their maximum diameter was not different between the two techniques (3.4 ± 0.3 µm with thin blood smear versus 3.3 ± 0.1 µm with MKT, *p*=0.92; Figure 3B and Table S3). On the MKT, microfilariae were not sheathed and had a blunt head with a relatively short head space, a compact column of nuclei beginning and ending with a single row, and a slender attenuated tail devoid of nuclei and sometimes ending in a button–hook curve. The length and morphology of the observed microfilariae were compatible with *Mansonella llewellyni* based on comparison with the reference slide and previously published literature [46–48] (Table 5). The location of the head space, nerve ring, and excretory pore as a percentage of body length was 1.2%, 21.7%, and 31.4%, respectively (Figure 3C).

## 4. Discussion

This investigation supports the interest in targeted *Wolbachia* qPCR gene amplification as a tool for filaria surveillance in the future. While the majority of sequences were *D. immitis*-associated *Wolbachia*, we also reported qPCR amplification of *Wolbachia* associated with one “Old World” zoonotic filarioid species in the United States (*D. repens*), and one wildlife filaria species (*M. llewellyni*) for which DNA sequences were not publicly available.

The establishment of *D. repens* in North America is a considerable risk for dog and human health, as both the host (dog) and vectors (*Anopheles*, *Aedes*, and *Culex* species) are present. For the present case, there is not sufficient evidence to determine if the *D. repens* infection was acquired in Europe, prior to importation of the dog to the United States, or in the United States. Reports of autochthonous infections in Florida and Mexico emphasize the potential for *D. repens* to establish in the Americas [14, 15]. In Europe, *D. repens* is the primary cause of subcutaneous and ocular dirofilariasis in humans and is among the fastest spreading zoonotic pathogens, with an increase in geographical range and increase in prevalence in established areas [49, 50]. Given that *D. immitis* antigen assays are not sensitive for detection of *D. repens* without heat treatment, MKTs or PCR based detection methods in canine and human populations should be implemented in the United States for surveillance purposes, particularly in imported animals [51]. *Wolbachia* has been repeatedly reported in *D. repens*-infected dogs in Europe [52, 53].

In this study, diagnostic amplification of *Wolbachia* DNA also facilitated the detection of *M. llewellyni* in raccoons. Given the lack of confirmatory DNA sequence or genomic data, the species identification was based on microscopic evaluation of microfilariae in raccoon blood collected prospectively. The previously published morphology (e.g., lack of sheath, blunt head, and curved tail devoid of nuclei) and mean length of *M. llewellyni* microfilariae were consistent with our findings. It is worth reinforcing that the technique used to visualize the parasites can impact morphometric characterizations, as shown here by a 21.6% increase in microfilaria length on MKT (287 µm) compared to blood smear (236 µm). Despite a high parasitemia in blood, microfilariae were not observed in 1 of 5 blood smears for two raccoons, highlighting the need to perform multiple blood smears or concentration methods (i.e., MKT) to decrease the risk of false-negative results. Given the concentration step, MKT has a greater sensitivity compared to blood smears [54]. In addition, MKT was required for precise morphology assessment.

Based on a PubMed search for “Mansonella llewellyni Wolbachia” and “Mansonella raccoon Wolbachia” yielding no results and review of the limited publications regarding *M. llewellyni* [24–29, 48], the authors believe this is the first study highlighting the presence of *Wolbachia* in *M. llewellyni*. *Wolbachia* have been identified in other *Mansonella* species, including *M. ozzardi* [31], *Mansonella perstans* [55], and *Mansonella perforate* and *Mansonella atelensis amazonae* [56]. Adult *M. llewellyni* are located in the raccoons' subcutaneous connective tissue and intermuscular fascia [27]. No parasites in these locations were found during the autopsy of the three filarial positive PCR raccoons. However, given their small number and size and not using a dissecting microscope, parasites could have been missed [27]. In addition, the presence of microfilariae without the observation of adults has also been reported [24, 27]. In the United States, microfilariae of *M. llewellyni* have been reported in several raccoons living in Florida [25], Georgia [24], Louisiana [22, 26], Maryland [27], and Tennessee [28] and in a single raccoon in North Carolina [29]. Our study demonstrated for the first time, to the best of our knowledge, *M. llewellyni*-infected raccoons in Kentucky, and additional cases in North Carolina. The ability of *M. llewellyni* to cause microscopic lesions was not assessed, as formalin-fixed tissues were not collected. Nevertheless, its pathogenicity in raccoons is likely low as there were no significant gross lesions in the PCR positive raccoons. Given the shared geography of *M. llewellyni* reservoir hosts (raccoons) and hematophagous arthropod vectors with humans and domestic animals, there is the potential for zoonotic or cross-species transmission but the authors did not locate any reports.

Human *Mansonella* infection is often asymptomatic but may manifest as acute swelling and itching of the skin, joint pain, or abdominal pain [57]. Successful elimination of *M. perstans* microfilaria in humans via doxycycline treatment targeting *Wolbachia* indicates the importance of future investigations involving *Mansonella-Wolbachia* interactions [36].

The detection of insect-associated *Wolbachia* species in the absence of detectable filaria DNA was unexpected. While *Wolbachia* are known to colonize a variety of insects, these *Wolbachia* strains are not regarded as infectious to mammalian species. None of the dogs had filarial DNA detected by PCR or were *D. immitis* antigen positive when tested by the NCSU–VBDDL. Interestingly, one dog was infected with *D. immitis* upon autopsy performed 15 days after blood collection and was *D. immitis* SNAP 4Dx Plus antigen positive at the home veterinary clinic. Injectable ivermectin and doxycycline was prescribed for treatment. Although unlikely given the slow rate of *Wolbachia* host switches, the detection of *Wolbachia* may be an undescribed strain infecting *D. immitis* or unrelated finding [58]. For all insect-associated *Wolbachia* dogs, DNA contamination was considered unlikely as these insect and *Wolbachia* species were not found in the NCSU–VBDDL during the study period, except for limited *C. felis* samples handled by different personnel. In the absence of detectable filarial infection, the ability of *Wolbachia* to survive in the mammalian host, invade mammalian cells, or survive in the absence of an arthropod or nematode host is unknown. Additional investigation, including sequential testing to determine if the infection persists, attempted isolation of *Wolbachia* from hosts with detectable insect-associated *Wolbachia* and experimental infection will be necessary to understand the potential role of *Wolbachia* as a mammalian pathogen.

Compared to SNAP 4Dx Plus Heartworm antigen detection (gold standard), detection of *D. immitis*-associated *Wolbachia* by amplification with the *Anaplasma/Ehrlichia* 16S rRNA qPCR boasted high specificity and NPV, but limited sensitivity (18%). While PPV was 88% utilizing SNAP 4Dx Plus Heartworm antigen detection as a gold standard, *D. immitis* DNA was detected in all samples with *D. immitis*-associated *Wolbachia* (100% PPV), suggesting the SNAP 4Dx Plus Heartworm antigen test does not detect all active *D. immitis* infections. This may be due to the delayed detection of the antigen test (~ 6 months after infection), limited detection of the antigen test (only female *D. immitis*), or imperfect sensitivity of antigen detection that is improved by heat treatment [59].

Limited research has attempted to utilize *Wolbachia* detection to diagnose filarial infection and generally report lower *Wolbachia* detection than filarial detection [60, 61]. Laidoudi et al. also reported the amplification of *D. immitis*-associated *Wolbachia* DNA in a majority of hosts with *D. immitis* DNA (75%, 9/12) as well as hosts without *D. immitis* DNA detected (29%, 8/29) [62]. In a survey of 307 domestic cats utilizing nested PCR targeting the *ftsZ* gene, *Wolbachia* was detected in 34 samples compared to filaria (*D. immitis*) detection in one sample [63]. The *Wolbachia* spp. reported in the present study were detected by non-specific amplification of the highly conserved bacterial 16S rRNA gene and had low sensitivity (18%) compared to the SNAP 4Dx Plus Heartworm antigen test. Therefore, qPCR for a highly sensitive and specific *Wolbachia* gene target, such as *ftsZ*, may increase diagnostic sensitivity for early or occult infections.

The lack of *Wolbachia* spp. detection in any additional animal species, specifically the domestic cat, was initially surprising as 3666 cats were tested by the *Anaplasma/Ehrlichia* qPCR during the study period. The domestic cat is documented to host *B. malayi*, *D. immitis*, and *D. repens*; however, only *D. immitis* infection is common in areas of the United States. *Dirofilaria immitis* infection in the cat requires a longer period of maturation and results in fewer adult parasites with a shorter lifespan compared to dogs [64]. Microfilaremia in the cat is typically transient with low microfilaria burdens, reducing the likelihood of detecting *Wolbachia* spp. by qPCR. *Dirofilaria immitis* infection is common in wild carnivores (e.g., coyote, fox), however the limited number of samples submitted for diagnostic testing was likely inadequate to detect a filaria infected host [65, 66]. The horse and donkey are host to multiple *Onchocerca* spp., however the location of microfilariae and adult filarioids in the skin, subcutaneous, or connective tissues prevents detection of *Wolbachia* DNA within the blood [67, 68].

Limitations of this study include not performing an MKT or PCR screening for filaria in all blood samples subjected to the *Anaplasma/Ehrlichia* 16S qPCR to assess the efficiency of *Wolbachia* detection. However, this was not possible due to the large number of samples tested by the VBDDL and practice of freezing samples at − 20°C after submission which is not compatible with the MKT. Furthermore, our inability to locate adult *Mansonella* from raccoons restricted phenotypic comparisons of adult morphology which is more thoroughly described in the literature [48]. The absence of longitudinal testing of animals prevented our ability to assess the persistence of *Wolbachia* in filarioid infected and uninfected hosts.

Based upon the findings in this study, there is a need to establish surveillance programs monitoring emerging and atypical filarioid species in humans, wildlife, and domestic animal species. Our findings expanded the spectrum of filarioid parasites colonized by *Wolbachia* to include *M. llewellyni*. We also for the first time published color images and genetic sequencing of *M. llewellyni*, a critical tool for species identification by parasitology and molecular laboratories. Surveillance programs for domestic animals and wildlife would help establish the diversity of filaria infecting the diverse species present in North America, as well as facilitate tracking their zoonotic potential. Our detection of *D. repens* in an imported dog reinforces the potential for *D. repens* to be introduced and establish in North America.

## Figures and Tables

**Table 1:** Adult and microfilaria localization of filaria infecting the domestic dog.

	Adult filaria niche	Microfilaria niche	Present in the United States	Reported Zoonotic potential	*Wolbachia* Infection
*Acanthocheilonema dracunculoides*	Skin	Blood	No	—	No
*Acanthocheilonema reconditum*	Skin	Blood	Yes	—	No
*Brugia ceylonensis*	Lymphatic system	Blood	No	—	Suspected
*Brugia malayi*	Lymphatic system	Blood	No	Yes	Yes
*Brugia pahangi*	Lymphatic system	Blood	No	—	Yes
*Brugia patei*	Lymphatic system	Blood	No	—	Suspected
*Cercopithifilaria bainae*	Skin	Blood	Yes	—	No
*Cercopithifilaria grassii*	Skin	Blood	No	—	No
*Dirofilaria immitis*	Pulmonary artery	Blood	Yes	Yes	Yes
*Dirofilaria repens*	Skin	Blood	No	Yes	Yes
*Onchocerca lupi*	Skin	Skin	Yes	Yes	Yes
*Thelazia californiensis*	Eye	Eye	Yes	—	No
*Thelazia callipaeda*	Eye	Eye	No	—	No

*Note:* Information extracted from the literature regarding filarial infection in the domestic dog including the adult and microfilaria niche, if the filarial species is found in the United States, if infection is considered zoonotic, and if *Wolbachia* is found infecting that filarial species. Multiple publications review canine filarial infection: [1–4].

**Table 2:** Demographic and clinicopathologic findings of dogs with insect-associated *Wolbachia* detected.

Detected *Wolbachia*	Age (years)	Breed	Sex	US State	Historical, clinical, and diagnostic findings
*Wolbachia pipientis* (*n* = 2)	2	Mixed breed	FC	IN	Weight loss, pancytopenia, and splenomegaly
	13	Labrador retriever	FC	TX	Megaesophagus, aspiration pneumonia, and increased ALP and ALT

*Ctenocephalides felis*-associated *Wolbachia* (*n* = 1)	1	German shepherd	FC	TX	Fever and endocarditis (treated with penicillin)

Nonspecific insect-associated *Wolbachia* (*n* = 2)	10	Siberian husky	MC	TX	Sprayed by skunk, Conjunctivitis progressing to anterior uveitis
	5	English setter	FI	TX	Sudden onset blindness, reverse sneezing, nasal malignant tumor with pulmonary metastases, and cardiac and pulmonary dirofilariasis

*Note:* The information was obtained from veterinary clinic records.

Abbreviations; C, castrated; F, female; I, intact; M, male.

**Table 3:** *Dirofilaria immitis*-associated *Wolbachia* 16S qPCR and SNAP 4Dx Plus results for dogs submitted to the NCSU–VBDDL.

		*Dirofilaria immitis* 4Dx SNAP Plus	
		Positive	Negative
*Dirofilaria immitis*-associated *Wolbachia* (16S qPCR)	Positive	37 (0.16%)	5 (0.02%)
	Negative	163 (0.71%)	22,881 (99.11%)

**Table 4:** Sensitivity, specificity, and predictive value of *Dirofilaria immitis*-associated *Wolbachia* detection in comparison to SNAP 4Dx Plus Heartworm and 28S Filarioid PCR gold standards.

	Gold standard	
	SNAP 4Dx Plus Heartworm (95% CI)	28S filarioid PCR (95% CI)
Number of samples	23,086	41

Sensitivity	18 (13–25)	N/A
Specificity	100 (100–100)	N/A
Positive predictive value (PPV)	88 (74–96)	100 (91–100)
Negative predictive value (NPV)	99 (99–99)	N/A

**Table 5:** Reported length and diameter of *Mansonella llewellyni* microfilariae.

Techniques (number of microfilariae observed)	Microfilariae length (range)	Microfilariae diameter (range)	Citation
Thin blood smear (*n* = 48)	236 (177–262)	3.4 (2.3–4.6)	This study
MKT (*n* = 57)	287 (261–321)	3.5 (2.8–5)	This study
2% formalin	288 (275–304)	4 (3–5)	[26]
MKT (*n* = 333)	270.2 (210–310)	2.5	[28]
Unreported	290 ± 5 (unknown)	2.5	[48]
Knott's test (*n* = 60)	286.5 ± 10.7 (st. dev.)	2.8 ± 0.2 (st. dev.)	[24]

*Note:* By default, range is reported in parentheses after metrics if available. If not available, the metrics reported by the citation is included and metric indicated in parentheses.

## Data Availability

The data that support the findings of this study are openly available in Dryad at https://doi.org/10.5061/dryad.bcc2fqzqp. Sequences are available on GenBank: *cox1* (PV258720-5), *myoHC* (PV269753-8), and *hsp70* (PV299582-6).

## Supporting Information

Supporting Information Table S1: Primers utilized for *Wolbachia* and filaria detection and phylogenetic comparison. Table S2: Total number of samples per animal species submitted for 16S *Anaplasma* and *Ehrlichi*a qPCR at the NCSU–VBDDL from 2017 to 2023, and number of samples with *Wolbachia* DNA detected by qPCR. Table S3: Detection of Microfilariae and Microfilariae Length and Maximum Diameter by Test.

## References

[1] Gruntmeir J., Kelly M., Ramos R. A. N., Verocai G. G. (2023). Cutaneous Filarioid Nematodes of Dogs in the United States: Are They Emerging, Neglected, or Underdiagnosed Parasites?. *Frontiers in Veterinary Science*.

[2] Perles L., Dantas-Torres F., Krücken J., Morchón R., Walochnik J., Otranto D. (2024). Zoonotic Dirofilariases: One, No One, or More than One Parasite. *Trends in Parasitology*.

[3] Sobotyk C., Foster T., Callahan R. T., McLean N. J., Verocai G. G. (2021). Zoonotic *Thelazia Californiensis* in Dogs From New Mexico, USA, and a Review of North American Cases in Animals and Humans. *Veterinary Parasitology: Regional Studies and Reports*.

[4] Orihel T. C., Eberhard M. L. (1998). Zoonotic Filariasis. *Clinical Microbiology Reviews*.

[5] Evans C. C., Greenway K. E., Campbell E. J. (2022). The Domestic Dog as a Laboratory Host for *Brugia Malayi*. *Pathogens*.

[6] Negron V., Saleh M. N., Sobotyk C., Luksovsky J. L., Harvey T. V., Verocai G. G. (2022). Probe-Based qPCR as an Alternative to Modified Knott’s Test When Screening Dogs for Heartworm (*Dirofilaria immitis*) Infection in Combination With Antigen Detection Tests. *Parasites and Vectors*.

[7] Otranto D., Brianti E., Dantas-Torres F. (2013). Species Diversity of Dermal Microfilariae of the Genus *Cercopithifilaria* Infesting Dogs in the Mediterranean Region. *Parasitology*.

[8] Sréter-Lancz Z., Széll Z., Sréter T. (2007). Molecular Genetic Comparison of *Onchocerca* sp. Infecting Dogs in Europe With Other Spirurid Nematodes Including *Onchocerca Lienalis*. *Veterinary Parasitology*.

[9] Simón F., Kramer L. H., Román A. (2007). Immunopathology of *Dirofilaria immitis* Infection. *Veterinary Research Communications*.

[10] Lammie P. J., Cuenco K. T., Punkosdy G. A. (2002). The Pathogenesis of Filarial Lymphedema. *Annals of the New York Academy of Sciences*.

[11] Wright I., Jongejan F., Marcondes M. (2020). Parasites and Vector-Borne Diseases Disseminated by Rehomed Dogs. *Parasites and Vectors*.

[12] Muñoz C., Gonzálvez M., Rojas A. (2020). Massive Microfilaremia in a Dog Subclinically Infected With *Acanthocheilonema Dracunculoides*. *Parasitology International*.

[13] Capelli G., Genchi C., Baneth G. (2018). Recent Advances on *Dirofilaria Repens* in Dogs and Humans in Europe. *Parasites and Vectors*.

[14] Hays K. M., Rodriguez J. Y., Little S. E. (2020). Heartworm Prevalence in Dogs Versus Cats: Multiple Diagnostic Modalities Provide New Insights. *Veterinary Parasitology*.

[15] Ramos-Lopez S., León-Galván M. F., Salas-Alatorre M., Lechuga-Arana A. A., Valencia-Posadas M., Gutiérrez-Chávez A. J. (2016). First Molecular Identification of *Dirofilaria Repens* in a Dog Blood Sample From Guanajuato, Mexico. *Vector-Borne and Zoonotic Diseases*.

[16] Dimzas D., Aindelis G., Tamvakis A. (2024). *Dirofilaria immitis* and *Dirofilaria Repens*: Investigating the Prevalence of Zoonotic Parasites in Dogs and Humans in a Hyperenzootic Area. *Animals*.

[17] Savić S., Stosi M. Z., Marcic D. (2020). Seroepidemiological Study of Canine and Human Dirofilariasis in the Endemic Region of Northern Serbia. *Frontiers in Veterinary Science*.

[18] Simón F., Muro A., Cordero M., Martin J. (1991). A Seroepidemiologic Survey of Human Dirofilariosis in Western Spain. *Tropical Medicine and Parasitology*.

[19] Ash L. R., Little M. D. (1964). *Brugia Beaveri* sp. n. (Nematoda: Filarioidea) From the Raccoon (*Procyon lotor*) in Louisiana. *The Journal of Parasitology*.

[20] Cleveland C. A., Eberhard M. L., Garrett K. B. (2020). *Dracunculus*, Species in Meso-Mammals from Georgia, United States, and Implications for the Guinea Worm Eradication Program in Chad, Africa. *Journal of Parasitology*.

[21] Harbut C. L., Orihel T. C. (1995). *Brugia Beaveri*: Microscopic Morphology in Host Tissues and Observations on Its Life History. *The Journal of Parasitology*.

[22] Smith J. L. (1980). Redescription of *Dipetalonema* (*Acanthocheilonema*) *Procyonis*, Price 1955 (Nematoda: Filarioidea) From the Raccoon. *The Journal of Parasitology*.

[23] Ramos R. A. N., Hakimi H., Salomon J. (2024). *Dirofilaria immitis* and *Dirofilaria Striata* (Spirurida: Onchocercidae) Detected in Wild Carnivores From Texas, United States. *International Journal for Parasitology: Parasites and Wildlife*.

[24] Pung O. J., Davis P. H., Richardson D. J. (1996). Filariae of Raccoons From Southeast Georgia. *The Journal of Parasitology*.

[25] Telford Jr. S. R., Forrester D. J. (1991). Hemoparasites of Raccoons (*Procyon lotor*) in Florida. *Journal of Wildlife Diseases*.

[26] Yates J. A., Lowrie R. C., Eberhard M. L. (1982). Development of *Tetrapetalonema Llewellyni* to the Infective Stage in *Culicoides hollensis*. *Journal of Parasitology*.

[27] Herman C. M., Price D. L. (1965). Epizootiologic Studies on Filarioids of the Raccoon. *The Journal of Wildlife Management*.

[28] Rabinowitz A. R., Patton S., Major V. (1985). Microfilariae of *Tetrapetalonema Llewellyni* in Raccoons of Cades Cove. *Journal of Wildlife Diseases*.

[29] Schaffer G. D., Davidson W. R., Nettles V. F., Rollor III. E. A. (1981). Helminth Parasites of Translocated Raccoons (*Procyon lotor*) in the Southeastern United States. *Journal of Wildlife Diseases*.

[30] Casiraghi M., Anderson T. J. C., Bandi C., Bazzocchi C., Genchi C. (2001). A Phylogenetic Analysis of Filarial Nematodes: Comparison With the Phylogeny of *Wolbachia* Endosymbionts. *Parasitology*.

[31] Casiraghi M., Favia G., Cancrini G., Bartoloni A., Bandi C. (2001). Molecular Identification of *Wolbachia* From the Filarial Nematode *Mansonella Ozzardi*. *Parasitology Research*.

[32] Hegde S., Marriott A. E., Pionnier N. (2024). Combinations of the Azaquinazoline Anti-*Wolbachia* Agent, AWZ1066S, With Benzimidazole Anthelmintics Synergise to Mediate Sub-seven-Day Sterilising and Curative Efficacies in Experimental Models of Filariasis. *Frontiers in Microbiology*.

[33] Landmann F., Voronin D., Sullivan W., Taylor M. J. (2011). Anti-Filarial Activity of Antibiotic Therapy Is Due to Extensive Apoptosis After *Wolbachia* Depletion From Filarial Nematodes. *PLoS Pathogens*.

[34] Brattig N. W., Bazzocchi C., Kirschning C. J. (2004). The Major Surface Protein of *Wolbachia* Endosymbionts in Filarial Nematodes Elicits Immune Responses Through TLR2 and TLR4. *The Journal of Immunology*.

[35] Hansen R. D. E., Trees A. J., Bah G. S. (2011). A Worm’s Best Friend: Recruitment of Neutrophils by *Wolbachia* Confounds Eosinophil Degranulation Against the Filarial Nematode Onchocerca Ochengi. *Proceedings of the Royal Society B: Biological Sciences*.

[36] Coulibaly Y. I., Demebele B., Diallo A. A. (2009). A Randomized Trial of Doxycycline for *Mansonella Perstans* Infection. *New England Journal of Medicine*.

[37] Manvell C., Berman H., Callahan B. (2022). Identification of Microbial Taxa Present in *Ctenocephalides felis* (cat Flea) Reveals Widespread Co-Infection and Associations With Vector Phylogeny. *Parasites and Vectors*.

[38] Altschul S. F., Gish W., Miller W., Myers E. W., Lipman D. J. (1990). Basic Local Alignment Search Tool. *Journal of Molecular Biology*.

[39] Livingston I. G., Gregory T. M., Hawkins E. C. (2024). Molecular Discovery of Filarial Nematode DNA in an Endangered Wild Pinniped (Galapagos Sea Lion, *Zalophus wollebaeki*). *Ecology and Evolution*.

[40] Moraes M. F. D., Pollo A. D. S., Hoppe E. G. L., Ionica A. M. (2022). Filarids (Spirurida: Onchocercidae) in Wild Carnivores and Domestic Dogs From the Brazilian Atlantic Forest. *PLOS Neglected Tropical Diseases*.

[41] Perles L., Otranto D., Barreto W. T. G. (2023). *Mansonella* sp. and Associated *Wolbachia* Endosymbionts in Ring-Tailed Coatis (*Nasua nasua*) in Periurban Areas From Midwestern Brazil. *International Journal for Parasitology: Parasites and Wildlife*.

[42] Stamatakis A. (2014). RAxML Version 8: A Tool for Phylogenetic Analysis and Post-Analysis of Large Phylogenies. *Bioinformatics*.

[43] Tyrrell J. D., Qurollo B. A., Mowat F. M., Kennedy-Stoskopf S. (2020). Molecular Prevalence of Detected Vector-Borne Organisms in Captive Red Wolves (*Canis Rufus*). *Journal of Zoo and Wildlife Medicine*.

[44] Zanfagnini L. G., Silva T. P. D., Campo D. R. (2023). Refrigerated Modified Knott Concentrate Enables Long-Term Morphological Viability of Canine Blood Microfilariae. *Brazilian Journal of Veterinary Medicine*.

[45] Chaltiel D., Hajage D. (2025). *Crosstable*: Crosstables for Descriptive Analyses. https://danchaltiel.github.io/crosstable/.

[46] Bain O., Mutafchiev Y., Junker K. (2015). Review of the Genus, *Mansonella*, Faust, 1929 Sensu Lato (Nematoda: Onchocercidae), With Descriptions of a New Subgenus and a New Subspecies. *Zootaxa*.

[47] Eberhard M. L., Orihel T. C. (1984). The Genus *Mansonella* (syn. *Tetrapetalonema*): A New Classification. *Annales de Parasitologie Humaine et Comparée*.

[48] Price D. L. (1965). Description of *Dipetalonema Interstitium* n. sp. From the Grey Squirrel and *Dipetalonema Llewellyni* n. sp. From the Raccoon. *Proceedings of the Helminthological Society of Washington*.

[49] Genchi C., Kramer L. H., Rivasi F. (2011). Dirofilarial Infections in Europe. *Vector-Borne and Zoonotic Diseases*.

[50] Sałamatin R., Pavlikovska T., Sagach O. (2013). Human Dirofilariasis Due to *Dirofilaria Repens* in Ukraine, an Emergent Zoonosis: Epidemiological Report of 1465 Cases. *Acta Parasitologica*.

[51] Sobotyk C., Savadelis M. D., Verocai G. G. (2021). Detection and Cross-Reaction of *Dirofilaria Repens* Using a Commercial Heartworm Antigen Test Kit. *Veterinary Parasitology*.

[52] Laidoudi Y., Ringot D., Watier-Grillot S., Davoust B., Mediannikov O. (2019). A Cardiac and Subcutaneous Canine Dirofilariosis Outbreak in a Kennel in Central France. *Parasite*.

[53] Sabūnas V., Radzijevskaja J., Sakalauskas P. (2019). *Dirofilaria Repens* in Dogs and Humans in Lithuania. *Parasites and Vectors*.

[54] Smith R. C., Tomlinson T. D., Bowles J. V., Starkey L. A. (2024). Comparative Performance Analysis of Different Microfilaria Testing Methods for *Dirofilaria immitis* in Canine Blood. *Parasites and Vectors*.

[55] Keiser P. B., Coulibaly Y., Kubofcik J. (2008). Molecular Identification of *Wolbachia* From the Filarial Nematode *Mansonella Perstans*. *Molecular and Biochemical Parasitology*.

[56] Ferri E., Bain O., Barbuto M. (2011). New Insights Into the Evolution of *Wolbachia* Infections in Filarial Nematodes Inferred From a Large Range of Screened Species. *PLoS ONE*.

[57] Ta-Tang T. H., Crainey J., Post R. J., Luz S. L., Rubio J. (2018). Mansonellosis: Current Perspectives. *Research and Reports in Tropical Medicine*.

[58] Sanaei E., Charlat S., Engelstädter J. (2021). *Wolbachia* Host Shifts: Routes, Mechanisms, Constraints and Evolutionary Consequences. *Biological Reviews*.

[59] Little S., Saleh M., Wohltjen M., Nagamori Y. (2018). Prime Detection of *Dirofilaria immitis*: Understanding the Influence of Blocked Antigen on Heartworm Test Performance. *Parasites and Vectors*.

[60] Bawm S., Khaing Y., Chel H. M. (2023). Molecular Detection of *Dirofilaria immitis* and Its *Wolbachia* Endosymbionts in Dogs From Myanmar. *Current Research in Parasitology and Vector-Borne Diseases*.

[61] Satjawongvanit H., Phumee A., Tiawsirisup S. (2019). Molecular Analysis of Canine Filaria and Its *Wolbachia* Endosymbionts in Domestic Dogs Collected From Two Animal University Hospitals in Bangkok Metropolitan Region, Thailand. *Pathogens*.

[62] Laidoudi Y., Marie J. L., Tahir D., Watier-Grillot S., Mediannikov O., Davoust B. (2020). Detection of Canine Vector-Borne Filariasis and Their *Wolbachia* Endosymbionts in French Guiana. *Microorganisms*.

[63] Turba M. E., Zambon E., Zannoni A., Russo S., Gentilini F. (2012). Detection of, *Wolbachia*, DNA in Blood for Diagnosing Filaria-Associated Syndromes in Cats. *Journal of Clinical Microbiology*.

[64] Simón F., Siles-Lucas M., Morchón R. (2012). Human and Animal Dirofilariasis: The Emergence of a Zoonotic Mosaic. *Clinical Microbiology Reviews*.

[65] Foster G. W., Main M. B., Kinsella J. M., Dixon L. M., Terrell S. P., Forrester D. J. (2003). Parasitic Helminths and Arthropods of Coyotes (*Canis latrans*) From Florida, U.S.A. *Comparative Parasitology*.

[66] Nelson T. A., Gregory D. G., Laursen J. R. (2003). Canine Heartworms in Coyotes in Illinois. *Journal of Wildlife Diseases*.

[67] Brown K. A., Johnson A. L., Bender S. J. (2023). *Onchocerca* sp. in an Imported Zangersheide Gelding Causing Suspensory Ligament Desmitis. *Journal of Veterinary Internal Medicine*.

[68] Radwan A. M., Ahmed N. E., Elakabawy L. M., Ramadan M. Y., Elmadawy R. S. (2016). Prevalence and Pathogenesis of Some Filarial Nematodes Infecting Donkeys in Egypt. *Veterinary World*.

