# Supplementary material for: Detection of Dirofilaria repens and Mansonella llewellyni in the United States by Wolbachia Surveillance
Source: Transbound Emerg Dis. 2025 Jul 21;2025:2778610. doi: 10.1155/tbed/2778610 (PMC12303656; doi:10.1155/tbed/2778610)
Supplement: Supporting Information — Table S1: Primers utilized for Wolbachia and filaria detection and phylogenetic comparison. Table S2: Total number of samples per animal species submitted for 16S Anaplasma and Ehrlichia qPCR at the NCSU–VBDDL from 2017 to 2023, and number of samples with Wolbachia DNA detected by qPCR. Table S3: Detection of Microfilariae and Microfilariae Length and Maximum Diameter by Test. [file 2778610.f1.docx]

**Supplemental Table 1 Primers utilized for *Wolbachia* and filaria detection and phylogenetic comparison.**

| Target Gene | Primer Name | Primer Sequence (5’-3’) | Citation |
| --- | --- | --- | --- |
| Filaria 28S | 28S-filaria-F | GTCCAATARSTGATGARGAAAC | (Livingston et al., 2024) |
|  | 28S-filaria-R | CTCACGGTACTTGTTTGCTATC |  |
| Filaria *cox1* | NTF | TGATTGGTGGTTTTGGTAA | (Casiraghi, Anderson, et al., 2001) |
|  | NTR | ATAAGTACGAGTATCAATATC |  |
| *Mansonella* *myoHC* | MyManF | GAAGCTGAG GCTCAAGCAAT | (Moraes et al., 2022) |
|  | MyManR | TCTGTTTTGCTCATCGCATT |  |
| *Mansonella hsp70* | h70ManF | TGAGACAGCTGGAGGTGTTATG | (Moraes et al., 2022) |
|  | h70ManR | ATCTTTCTGTGCCTCATCATCTG |  |
| *Wolbachia* 16S | AE16S_45F | AGCYTAACACATGCAAGTCGAACG | (Tyrrell et al., 2020) |
|  | AE16S_299R | CCTCTCAGACCAGCTATAGATCA |  |

**Supplemental Table 2 Total number of samples per animal species submitted for 16S *Anaplasma* and *Ehrlichi*a qPCR at the NCSU-VBDDL from 2017-2023, and number of samples with *Wolbachia* DNA detected by qPCR.**

| **Animal Species** | **Number of Samples Tested** | **Number of samples with *Wolbachia* DNA (% Total Samples)** |
| --- | --- | --- |
| Canine | 35,179 | 57 (0.16%) |
| Feline (Small Animal) | 3,666 | 0 (0%) |
| Equine | 338 | 0 (0%) |
| Rhinoceros | 140 | 0 (0%) |
| Raccoon | 40 | 3 (7.50%) |
| Antelope | 29 | 0 (0%) |
| Caprine | 24 | 0 (0%) |
| Reindeer | 11 | 0 (0%) |
| Bovine | 8 | 0 (0%) |
| Tiger | 8 | 0 (0%) |
| Porcine | 6 | 0 (0%) |
| American Black Bear | 5 | 0 (0%) |
| Coyote | 5 | 0 (0%) |
| Lion | 5 | 0 (0%) |
| Otter | 5 | 0 (0%) |
| Cheetah | 4 | 0 (0%) |
| Giraffe | 4 | 0 (0%) |
| Llama | 4 | 0 (0%) |
| Deer | 3 | 0 (0%) |
| Primate | 3 | 0 (0%) |
| Rabbit | 3 | 0 (0%) |
| Red Fox | 3 | 0 (0%) |
| Alpaca | 2 | 0 (0%) |
| Arctic Fox | 2 | 0 (0%) |
| Bovine Exotic | 2 | 0 (0%) |
| Elk | 2 | 0 (0%) |
| Unknown Large Feline | 2 | 0 (0%) |
| Gazelle | 2 | 0 (0%) |
| Unknown Animal | 2 | 0 (0%) |
| Unknown Exotic | 2 | 0 (0%) |
| Unknown Primate | 2 | 0 (0%) |
| * | 1 | 0 (0%) |

The animal species was reported by the owner or veterinary staff upon submission. * Only a single sample was collected from the Bobcat, Camel, Emu, Ferret, Gibbon, Guinea Pig, Harbor Seal, Hyena, Leopard, Maned Wolf, Orangutan, Pine Martin, Polar Bear, Porcupine, Ring Tailed Lemur, Ruffer Lemur, Sloth, Whale, Wolf, and Woodchuck.

**Supplemental Table 3 Detection of Microfilariae and Microfilariae Length and Maximum Diameter by Test**

| Raccoon (Sex) |  | Modified Knott’s Test | | | | Thin blood smear | | | |
| --- | --- | --- | --- | --- | --- | --- | --- | --- | --- |
|  | Molecular Result (28S Filarial PCR) | Result | Length  (range) | Max Diameter  (range) | Number per slide (range) | Result^a^ | Length (range) | Max Diameter (range) | Number per slide (range) |
| R1 (M) | - | - | N/A | N/A | 0 | - (5/5) | N/A | N/A | 0 |
| R2 (F) | - | - |  |  | 0 | - (5/5) |  |  | 0 |
| R3 (M) | + | + | 281 (261-309) | 3.5 (3.2-3.9) | 62-97 | + (5/5) | 200 (177-232) | 3.3 (2.8-3.7) | 2-8 |
| R4 (M) | + | + | N/P | N/P |  | + (4/5) | 241 (214-261) | 3.4 (2.3-4.6) | 0-25 |
| R5 (F) | + | + | 290 (271-322) | 3.5 (2.8-5) | 193-221 | + (4/5) | 229 (198-262) | 3.0 (2.5-3.7) | 0-3 |

^a^ Number of blood smears with that result

N/A, not available as microfilariae were not observed

N/P, not performed due to insufficient blood

M – male, F – female
